# Efficient Scheduling of Scientific Workflows with Energy Reduction Using Novel Discrete Particle Swarm Optimization and Dynamic Voltage Scaling for Computational Grids

**DOI:** 10.1155/2015/791058

**Published:** 2015-05-14

**Authors:** M. Christobel, S. Tamil Selvi, Shajulin Benedict

**Affiliations:** ^1^Ponjesly College of Engineering, Nagercoil, Tamil Nadu 629003, India; ^2^National Engineering College, Kovilpatti, Tamil Nadu 628503, India; ^3^HPCCloud Research Laboratory, St. Xavier's Catholic College of Engineering, Chunkankadai, Tamil Nadu 629003, India

## Abstract

One of the most significant and the topmost parameters in the real world computing environment is energy. Minimizing energy imposes benefits like reduction in power consumption, decrease in cooling rates of the computing processors, provision of a green environment, and so forth. In fact, computation time and energy are directly proportional to each other and the minimization of computation time may yield a cost effective energy consumption. Proficient scheduling of Bag-of-Tasks in the grid environment ravages in minimum computation time. In this paper, a novel discrete particle swarm optimization (DPSO) algorithm based on the particle's best position (pbDPSO) and global best position (gbDPSO) is adopted to find the global optimal solution for higher dimensions. This novel DPSO yields better schedule with minimum computation time compared to Earliest Deadline First (EDF) and First Come First Serve (FCFS) algorithms which comparably reduces energy. Other scheduling parameters, such as job completion ratio and lateness, are also calculated and compared with EDF and FCFS. An energy improvement of up to 28% was obtained when Makespan Conservative Energy Reduction (MCER) and Dynamic Voltage Scaling (DVS) were used in the proposed DPSO algorithm.

## 1. Introduction

Grid is an environment mainly designed for the dynamic computing applications of the scientific world. By definition, grid is a type of parallel and distributed system that permits sharing, selecting, and accumulation of geographically distributed independent resources dynamically at runtime reliant on their availability, capability, performance, budget, and user's quality of service requirements. Based on the historical view grid is designed mainly for massive computation, but the present trend is using thousands of computers to search for an extraterrestrial intelligence using SETI@home project [1]. During these centuries, computing equipment has become a part of life from kids to grown-ups and each piece of equipment may have a built-in processor, which may be idle or busy all over the clock cycles. Utilizing the idle clock cycles in the grid may reduce the computation time of the scientific applications. So scheduling of jobs within the grid is likely a key contest of the grid system. Scheduling refers to allocating jobs to the processors in an efficient manner to meet user defined constriants.

Recent schedulers are designed to optimise these constraints to a greater extent. Since present, nature inspired algorithms like ant colony optimization (ACO), particle swarm optimization, intelligent water drop algorithm, bee colony optimization [2], and so forth provide promising outcome when compared to other scheduling algorithms, as grid resources are widely distributed, grid scheduling algorithms should also be able to traverse through higher dimensions. Efficient use of processor cycles using a scheduling algorithm has a bigger impact in the reduction of computation time of the processors. Since grid resources are dynamic and diverse in nature, scheduling algorithms should also exhibit these properties.

In addition, a grid resource may be either of these cases, a super computer with more number of processors or computing processors distributed geographically. Subsequently each processing unit needs power; there comes another concern, the energy consumption of the computing processors. Increase in energy consumption leads to loss in the form of heat, which results in higher cooling rates and decrease in reliability of the computing resources. Any system is graded based on its performance; the higher the speed of the computing processor, the higher the performance of the grid computing system, which takes the lead towards enormous energy consumption. So technologies are striving to have better performance in the grid system in both minimum computation time and energy consumption.

## 2. Related Work

Any type of application involves scheduling for its better performance. In grid, scheduling is considered as* NP*-hard, so lots of scheduling algorithms have been proposed in the literature. Scheduling refers to allocation of dynamically arising jobs onto the computing processors, satisfying any objective function with any user defined constraints. Metaheuristics like genetic algorithm, particle swarm optimization, simulated annealing, threshold accepting algorithm, ant colony optimization, and so forth were implemented for the grid scheduling problem. A novel particle swarm optimization technique was developed for efficient resource allocation based on comprehensive learning strategy to prevent premature convergence and to improve the solution [3]. Also various versions of chemical reaction optimization algorithm, which is a population based method, are inspired by the interaction between molecules in a chemical reaction that performs better for large scale applications [4].

A discrete particle swarm approach for the grid scheduling problem aiming to minimize makespan and flow time simultaneously was proposed [5]. For combinatorial optimization problems like travelling salesman problem and the multidimensional knapsack problem a novel set based PSO method was proposed and promising results were obtained [6]. Look-ahead genetic algorithm was proposed for optimizing both reliability and makespan of the workflow applications [7]. Also a comparative study of PSO and ACO was performed and the study concludes that PSO has better performance compared to ACO for grid job scheduling [8]. Mostaghim et al. [9] suggested a multiobjective particle swarm optimization for the modern grid computing platforms. Coutinho et al. [10] propose the HGreen heuristic (heavier tasks on maximum green resource) for workflow scheduling on global grids.

Today energy consumed by the computing processors has become one of the top stories of the planet. A small reduction in energy consumption per hour per day may lead to sufficient energy reduction all over the year and so recent schedulers are aiming to minimize energy in addition to other user defined objectives. Kołodziej et al. [11] address a global minimization problem with makespan and energy consumption as the main objective. They have used dynamic voltage and frequency scaling model for the management of the cumulative energy utilized by the grid resources and genetic algorithm for scheduling. Wang et al. [12] employ dynamic voltage frequency scaling for reducing power consumption of parallel tasks in a cluster along with some green service level agreement. Lee and Zomaya [13] proposed two scheduling algorithms, namely, Energy Conscious Scheduling (ECS) and ECS + idle, using DVS at the expense of sacrificing clock frequencies. Also two novel power-aware scheduling algorithms based on slack reclamation technique for multiprocessor real-time systems with the assumption of voltage and speed adjustment overhead being negligible were proposed [14].

Peng et al. [15] use a digital instruction cycle based dynamic voltage scaling (iDVS) power management strategy with adaptive instruction cycle control scheme for low power digital signal processor and obtained power saving of about 53%. Lindberg et al. [16] compared and analysed seven heuristic based greedy energy efficient scheduling algorithms for computational grids with a conclusion; for small-sized problems, algorithms like Greedy-min and Greedy-Deadline perform best and, for large-sized problems, ObFun algorithm performs better in terms of mean energy consumption and mean makespan compared to other proposed heuristics. Kim et al. [17] presented a power-aware scheduling of Bag-of-Tasks with dynamic voltage technique with both time-shared and space-shared resource sharing policies in a cluster. Albers [18] gives the survey of energy efficient algorithms on both the system and the device level.

The rest of the paper is described as follows.  Section 3 briefly describes the grid scheduling problem.  Section 4 gives a short note on DPSO algorithm and Section 5 explains the proposed discrete particle swarm optimization (DPSO) algorithm.  Section 6 illustrates energy aware scheduling with dynamic voltage scaling (DVS) technique and Section 7 briefly gives results and discussion. Conclusion is given in Section 8.

## 3. Problem Description

**Figure 1:**
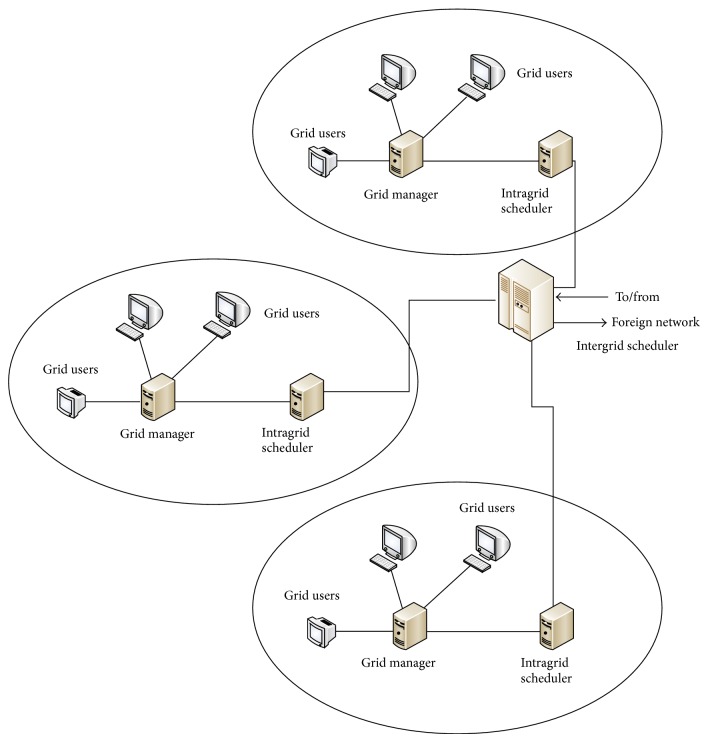
Intergrid scheduler architecture.

Grid is an environment of vast computing resources available on demand, based on user's requirements. The user's requirement may vary based on their objectives like computation cost, energy consumption, makespan, flow time, quality of service, and so forth; Figure 1 shows the intergrid scheduler architecture implemented in TIFAC core engineering [19].

The major components of intergrid scheduler architecture are described below.

*Grid Users*. Grid users submit their jobs in the form of workflows to the local grid managers.

*Grid Managers*. The workflows submitted by the grid users are collected by the grid managers and fed as a request for scheduling to the intragrid scheduler.

*Intragrid Scheduler*. The updated information about the idle resources at time “*t*” is gathered by the intragrid scheduler and the workflows are scheduled within the deadline.

*Intergrid Scheduler*. If the resources needed for scheduling the jobs are geographically distributed then there is an indispensability of intergrid schedulers.

In order to increase the performance of the grid system, each subsystem should be designed to produce the best output. The functional building block consists of resource discovery, secure access, resource allocation, fault tolerant detection, data management, communications, and so forth [20]. Of these, the resource allocation system can perform better by allocating the jobs to the available resources, satisfying the objective imposed by the user. Today as the resources are distributed worldwide, the scheduling algorithm should be capable of finding the best solution by traversing through large dimensions. The scheduling problem is mapping “*N*” number of jobs to “*P*” number of processors. Here the workflow model is assumed to be DAG (Directed Acyclic Graph). Let us define the objective of minimizing the makespan of the schedule:

(1) Makespan=max⁡⁡∑i=1PCi,cti,j=communication time between the jobs  i  and  j,∀i≠jcomputation time of the  ith  job,∀i=j.

**Example 1 .**

**Figure 2:**
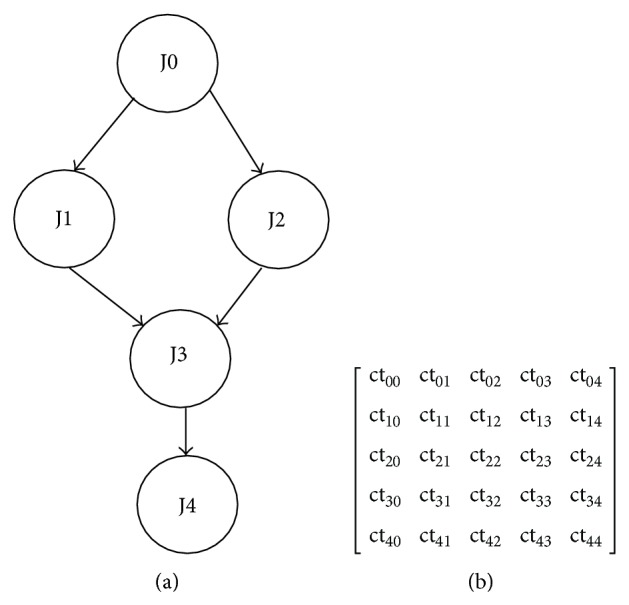
(a) DAG workflow model. (b) Matrix representation of computation and communication time.

**Figure 3:**
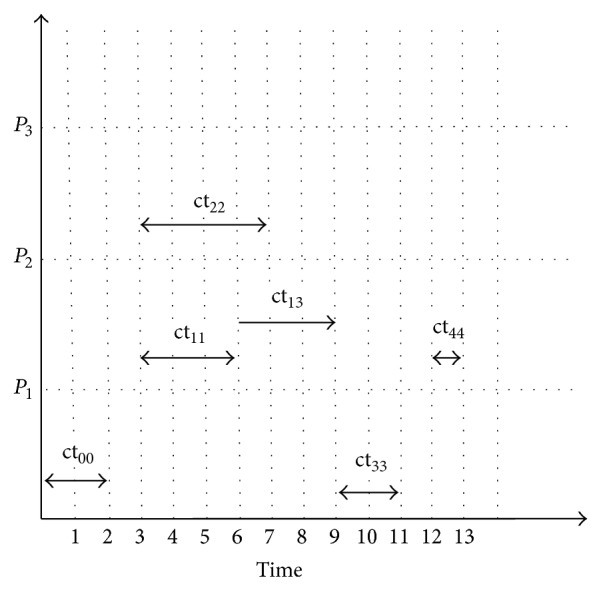
Scheduling of workflows for Example 1.

Consider that we have number of processors equal to three and number of jobs equal to five with the computation and communication time given for the workflow graph shown in Figure 2(a). The diagonal value in Figure 2(b) corresponds to the computation time of each node and the upper triangle gives the communication cost of the corresponding node with other nodes in the workflow graph. For a single workflow, the scheduling of jobs onto the processor is shown in Figure 3. Here *P*_1_, *P*_2_, and *P*_3_ are computing processors and ct_00_ and ct_11_ are the computation time of job node J0 and job node J1, respectively, with ct_13_ being the communication time between J1 and J3. The computation matrix is given below:

(2) $$\begin{array}{c} {\text{ct}}_{5,5}=\left[\begin{array}{ccccc} 2 & 1 & 2 & 0 & 0\\ 0 & 3 & 0 & 3 & 0\\ 0 & 0 & 4 & 1 & 0\\ 0 & 0 & 0 & 2 & 1\\ 0 & 0 & 0 & 0 & 1 \end{array}\right]. \end{array}$$

Grid computing applications like SETI@home, drug discovery, high energy physics, and so forth require hundreds or thousands of jobs to be done on the computing processors, so we are in need of a scheduling policy to increase the performance of the system based on users' or customers' request. The first and the foremost parameter to be optimized is the makespan, which is the application completion time, to be minimized. The scheduling problem is defined with certain assumptions.

- Each workflow is represented as a Directed Acyclic Graph, with jobs executed in the ordered series after the completion of the previous job, and parallel jobs can be executed in two different processors.
- The workflow jobs are considered as abstract model in which each and every job can be assigned to any of the available grid resources.
- Time is considered as the QoS constraint at the workflow level.
- With the static resources available, the dynamic workflows are to be scheduled at the run time with no preemption of tasks.

## 4. PSO Algorithm

PSO algorithm is a heuristic algorithm based on bird flocking and fish schooling [21]. Due to the simplicity of the algorithm, it is used widely in many optimization problems like travelling salesman problem, knapsack problem, permutation flow shop problem, grid scheduling problem, team orienteering problem, capacitated vehicle routing problem, and so forth [22–24]. For the grid scheduling problem discrete particle swarm optimization performs better when scanning through the vast environment.

### 4.1. Discrete Particle Swarm Optimization (DPSO) Algorithm

(i) Initialize the particles position, velocity, particles personal best, and particles global best vectors depending on the dimension of the problem (Table 2).
(ii) Initialize the value of inertia weight, social cognitive coefficients, swarm size, and the number of iterations to be performed (Table 1).
(iii) Model the fitness function based on the user's objective.
(iv) Calculate the fitness function for each particle in the swarm and select the best fitness based on the objective whether to minimize or maximize the function.
(v) Update the velocity vector using the following formula:
  (3) Vet+1=w×Vet+R1×C1×Pbest−Po+R2×C2×Gbest−Po.
 Po is position of a particle.
 
*P*_best_ is personal best solution.
 
*G*_best_ is global best solution.
 
*w* is inertia.
 
*C*_1_ is particle increment.
 
*C*_2_ is global increment.
 
*R*_1_, *R*_2_ are uniform random numbers between 0 and 1.
 Ve^*t*^ is velocity of the particle at time “*t*.”
(vi) Update the position vector of the particle using the following formula:
  (4) $$\begin{array}{c} {\text{Po}}^{t+1}={\text{Po}}^{t}+{\text{Ve}}^{t+1}. \end{array}$$
(vii) Calculate the fitness function for the new set of population and continue until the number of iterations is met or satisfying solution is obtained.

## 5. Novel DPSO

As grid resources are distributed geographically and the job request to these resources also has increased tremendously by time, the dimensionality of the scheduling problem increases. This increase in dimension decreases the performance of the DPSO algorithm, since the particles positions overlap each other after updating the particles position vector. To overcome this we propose a novel DPSO algorithm, where, after velocity updating, the position of the particle is updated based on two features; one is based on the particles personal best (pbDPSO) and the other is based on particles global best (gbDPSO) position. The Pseudocode for the proposed DPSO is given in Algorithm 1, while the pseudocodes for pbDPSO and gbDPSO are given in Algorithms 2 and 3.

The population now consists of only feasible solution after updating the position using the proposed updating procedure. In pbDPSO based updating, the new position of the particle is updated based on (3) and (4) and if the solution is not feasible it is updated using the particles personal best solution.

**Figure 4:**
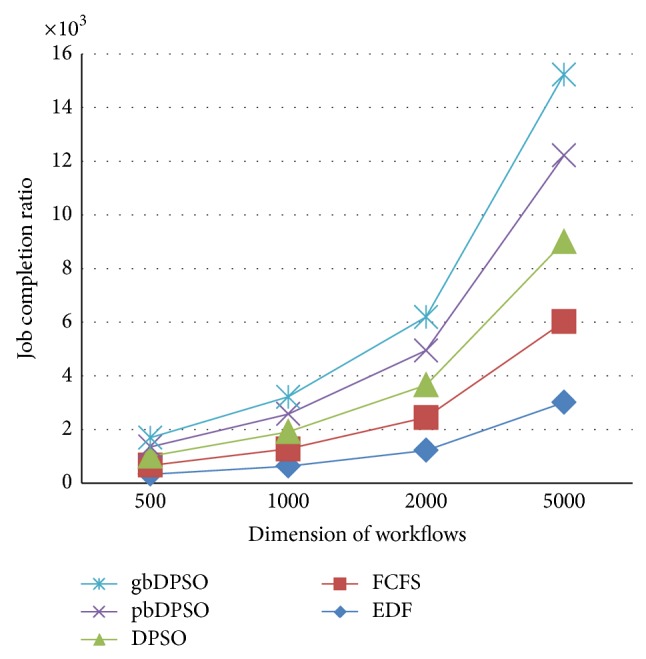
Comparison of job completion ratio using novel DPSO algorithm.

**Figure 5:**
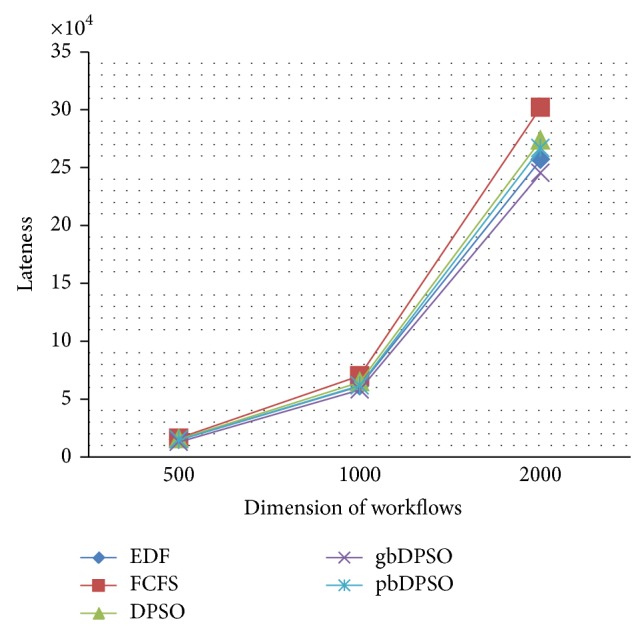
Comparison of lateness using novel DPSO algorithm.

In gbDPSO, particle is updated with the knowledge of both velocity and particles global best solution, if the updated solution using (3) and (4) is infeasible. The job completion ratio is defined as the ratio of number of workflows completed within the deadline to the total number of workflows, and lateness is the time required to complete the job beyond its deadline. Figures 4 and 5 show the comparison of job completion ratio and lateness in arbitrary time units, respectively. From the graph shown in Figure 4, it is clear that gbDPSO based algorithm outperforms pbDPSO and other algorithms. Also lateness of the job is minimum for gbDPSO compared to pbDPSO based algorithm, which is understood from Figure 5.

## 6. Energy Aware Scheduling

Energy consumption of the computing processors has become a research topic in the heterogeneous computing environment [25]. In specific, distributed computing environment usually consists of any number of supercomputers, servers, and large databases and thousands of personal computers.

In whole we can conclude that distributed computing environment is mainly composed of processors and so a small amount of minimization in energy consumption using any algorithm or any other techniques may upswing the performance of the computing environment. Energy can be minimized in both hardware level and software level. Dynamic voltage and frequency scaling are one of the standard techniques espoused for energy consumption in the hardware level. In the software level, an efficient scheduling scheme with constraints like energy and computation time has to be implemented in the scheduler for minimizing the energy consumption [11]. The power consumption of the processor for executing a job can be calculated using the following formula:

(5) $$\begin{array}{c} \text{power consumed Pc}=S*C*v*v*f, \end{array}$$

where *S* denotes the number of switches per clock cycle, *C* represents the capacitive load, and *v* and *f* represent the voltage and frequency of the computing processor. Assuming that switches per clock cycle and capacitive load are constant, the power consumption now depends only on the voltage and frequency of the processor. So the energy consumption of a processor *P*_*j*_ for executing a job JR_*i*_ can be calculated as follows:

(6) EcPc∗Tk,

(7) Ec=α∗v∗v∗f∗Tk,

where

(8) Tk=∑k=1Pcomputation time of Processor  Pk,α=S∗C,nk⟶total number of jobs executed on processor  Pj.

From (6) energy consumption also depends on the computation time of the processors and, so, efficient reduction in computation time yields a considerable reduction in energy consumption. From Table 4, it is clear that the proposed algorithm considerably reduces the energy consumed by the processors.

### 6.1. DVS Enabled Scheduling

The percentage of energy reduction can be increased by using dynamic voltage and frequency scaling in addition to energy aware scheduling. DVS is scaling down of processor frequency so that the processor voltage is reduced resulting in a significant energy consumption.  Table 5 shows the voltage/frequency for a single machine class. While lowering the processor frequency the computation time of the particular job running on the processor increases and so, for our scheduling problem, the frequency of the computing processor is scaled down for jobs that are having idle time between the executions of two tasks without any reduction in the makespan of the schedule. An improvement in energy consumption can be achieved using MCER [13] in addition to DVS, with the makespan unaltered:

(9) Total energy consumed  ETOTAL=ECOMPUTATION+EIDLE,

(10) ECOMPUTATION=α×∑k=1P ∑i=1nkCk,i×Vslevelk,i2×Fslevelk,i,

(11) Vslevelk,i=vmax⁡∀fk,i+1=sk,i+1vsl∀fk,i+1≠sk,i+1,

(12) EIDLE=∑k=1PMakespan−Completionk×vmin⁡2×fmin⁡,

(13) Energy Reduction in  %=Δ1−Δ2Δ2×100%.

**Figure 6:**
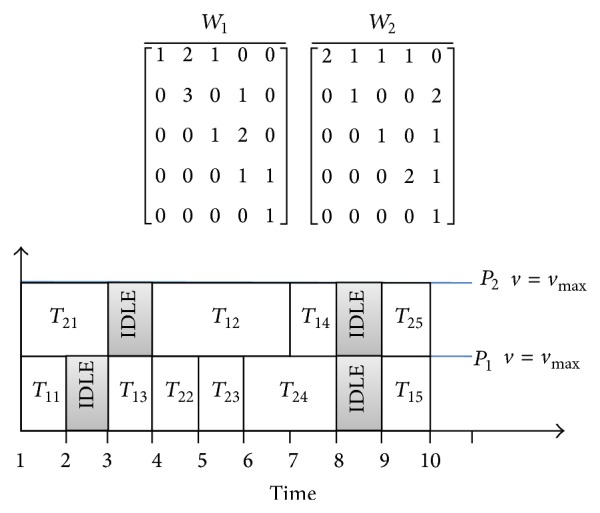
Scheduling of workflows *W*_1_ and *W*_2_.

**Figure 7:**
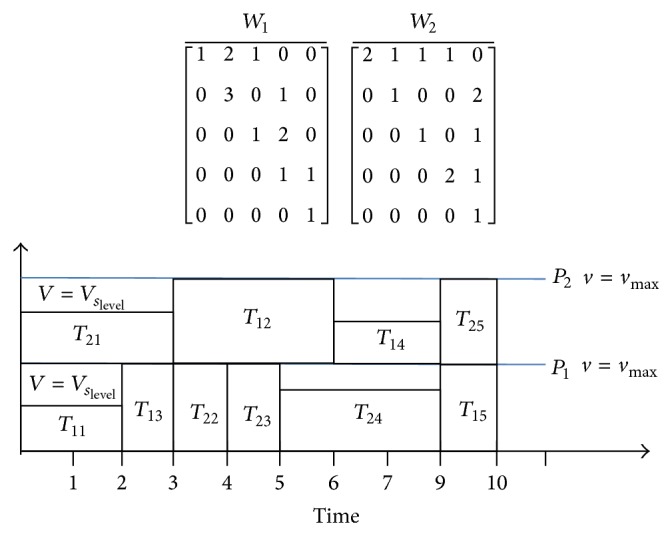
Scheduling of *W*_1_ and *W*_2_ using DVS.

Δ_2_ is energy consumed using novel DPSO with DVS and MCER, Δ_1_ is energy consumed using EDF or FCFS or DPSO, *n*_*k*_ is the number of jobs in the *k*th processor and *C*_(*k*,*i*)_ denotes the computation time of *i*th job on *k*th processor. All processors are assumed to be on DVS mode with *V*_*s*_level(*k*,*i*)__ denoting the subset of supply voltage level, for the *i*th job running on the *k*th processor, and *f*_*s*_level(*k*,*i*)__ being the frequency level of *i*th job running on the *k*th processor. Each job scheduled to the processor is assumed to have a start and finish time and let us denote *s*_(*k*,*i*)_ and *f*_(*k*,*i*)_ as the start and finish time of *i*th job running on the *k*th processor, respectively. The energy consumed by the processor is minimized using DVS without any increase in makespan as a constraint. Consider an example of scheduling two workflows, and each workflow is represented as DAG workflow model. The computation time and the communication time are given in the matrix form. *T*_*ij*_ denotes the computation time of *j*th task in *i*th workflow. IDLE in Figure 6 denotes the time, when the processor is idle. *V*_*s*_level__ in Figure 7 gives the supply voltage from a given set, for the DVS enabled processors.

**Figure 8:**
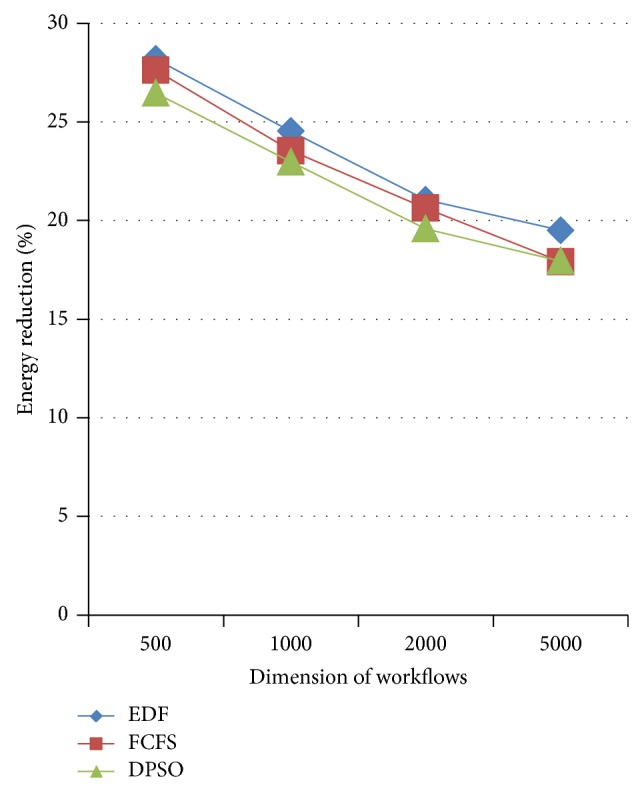
Energy reduction of novel DPSO with DVS and MCER compared to EDF, FCFS, and DPSO algorithms.

Also it is obvious from Figure 7 that the makespan of the schedule is unaltered with a decrease in processor speed of certain tasks resulting in the reduction of energy consumption.  Table 6 shows the energy consumed by the DVS enabled processors using novel DPSO algorithm. DVS enabled scheduling thus provides a significant energy reduction. The amount of energy reduction using novel DPSO with DVS and MCER is compared to EDF, FCFS, and DPSO using (13) and is shown in Figure 8.

## 7. Results and Discussion

The experiments are simulated in Java. From Table 3, it is obvious that makespan is reduced to a considerable level compared to EDF, FCFS, and DPSO. Since energy consumed by the processors is directly proportional to the computation time, an efficient reduction in energy consumption is obtained with the decrease in makespan using pbDPSO and gbDPSO. Subsequently, energy depends on voltage and frequency of the computing processors, and so any decrease in voltage may further reduce energy. In this grid scheduling problem, DVS enabled scheduling with MCER is adopted to further reduce energy by lowering the voltage of the computing processors for certain workflows without any increase in makespan of the schedule. From the results, an efficient reduction in energy is obtained, which is clear from Table 6 and Figure 8.

## 8. Conclusion

In this paper, we have referred to the scheduling problem in a grid environment. To minimize the makespan, a novel DPSO is proposed based on the particles personal best and global best. The proposed algorithm performs best when spanning through vast dimensions of grid. Also the energy of the computing processors is minimized using the proposed algorithm with dynamic voltage scaling and MCER without any increase in the makespan of the schedule.

## Figures and Tables

**Algorithm 1:**
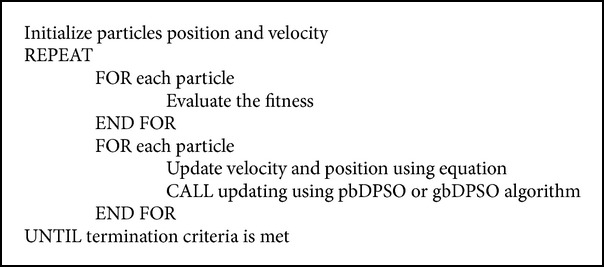


**Algorithm 2:**
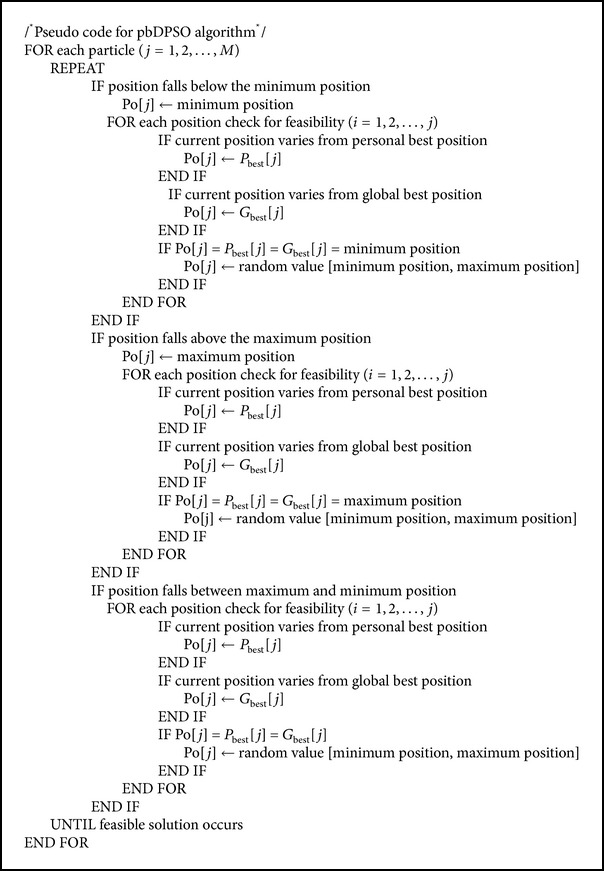


**Algorithm 3:**
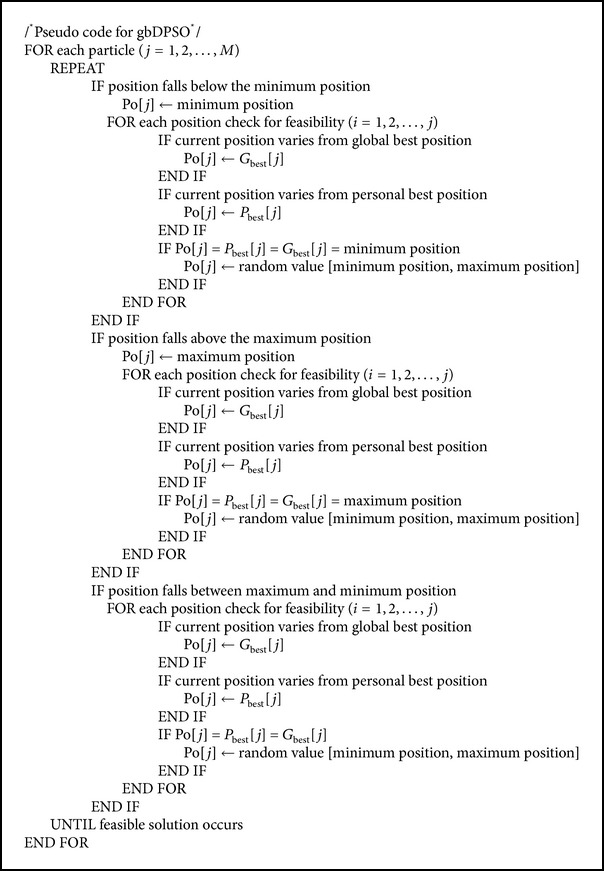


**Table 1:** PSO parameter setting.

Swarm size	30
Iterations	20
Inertia	0.9
*C*_1_, *C*_2_	[1, 2]

**Table 2:** Initial settings for scheduling.

Dimension of workflows	Number of workflows/number of processors
Small	500/21
Medium	1000/21
Large	2000/21
Very large	5000/21

**Table 3:** Makespan values in arbitrary time units.

Algorithms	Dimension of workflows			
	Small	Medium	Large	Very large
FCFS	445	873	1739	4294
EDF	447	880	1745	4352
DPSO	441	869	1724	4295
pbDPSO	436	866	1721	4288
gbDPSO	436	864	1719	4283

**Table 4:** Energy consumed by the processors in joules.

Dimension of workflows	Algorithms				
	FCFS	EDF	DPSO	pbDPSO	gbDPSO
Small	21026.25	21120.75	20837.25	**20601.0**	20601.0
Medium	41249.25	41580	41060.25	40918.5	**40824.0**
Large	82167.75	82451	81459.0	81317.25	**81222.75**
Very large	202891.5	205632	202938.75	202608.0	**202371.75**

**Table 5:** Voltage and frequency levels for a single machine class.

Level	Voltage	Relative frequency
0	1.5	1.0
1	1.4	0.9
2	1.3	0.8
3	1.2	0.7
4	1.1	0.6
5	1.0	0.5
6	0.9	0.4

**Table 6:** Energy consumed by the computing processors in joules.

Dimension of workflows	Algorithms			
	EDF	FCFS	DPSO	Novel DPSO with DVS and MCER
Small	21120.75	21026.25	20837.25	**16476.71**
Medium	41580.0	41249.25	41060.25	**33389.31**
Large	82451.75	82167.75	81459.0	**68118.27**
Very large	205632.0	202891.5	202938.75	**172064.16**
